# Research progress of ferroptosis in gynecological diseases

**DOI:** 10.1080/07853890.2026.2688328

**Published:** 2026-06-19

**Authors:** Wen Chen, Wencheng Zhou, Songjun Liu

**Affiliations:** a Tongde Hospital of Zhejiang Province Affiliated to Zhejiang Chinese Medical University (College of Integrated Traditional Chinese and Western Medicine Clinical Medicine), Hangzhou, China; b The First Affiliated Hospital of Zhejiang Chinese Medical University (Zhejiang Provincial Hospital of Chinese Medicine), Hangzhou, China; c Department of Gynecology, Tongde Hospital of Zhejiang Province, Hangzhou, China

**Keywords:** Ferroptosis, endometrial cancer, endometriosis, ovarian cancer

## Abstract

**Background:**

The concept of ferroptosis debuted as a newly defined programmed cell death in 2012. Among programmed cell death mechanisms, ferroptosis stands out as being fundamentally dependent on iron. At the heart of this mechanism lies the progressive accumulation of lipid peroxides – a chain reaction propelled by available iron, terminating when intracellular levels become fatally toxic. Inhibition of cystine transporters within cells (notably induced by compounds like Erastin) initiates a chain reaction: when intracellular levels of glutathione (GSH) become depleted, downstream suppression of *glutathione peroxidase 4 (GPX4)* activity impedes lipid peroxide clearance, whose accumulation drives the cell toward death upon exceeding a critical concentration.

**Discussion:**

Early-stage experimental models highlight ferroptosis’s contribution to propelling high-impact gynecological disease progression, namely precancerous endometrial hyperplasia, endometrial cancer (EC), endometriosis (EMS), and ovarian cancer (OC). Hence, elucidating the intricate regulatory machinery behind ferroptosis in gynecological pathologies bears both theoretical importance and translational promise.

**Conclusion:**

This review aimed to systematically synthesize current knowledge on ferroptosis in gynecological diseases and their associated regulatory mechanisms, offering insights relevant to both basic research and clinical application.

## HIGHLIGHTS

- The article may have systematically linked ferroptosis with various gynecological diseases for the first time, revealing both commonalities and differences in the regulatory networks of ferroptosis across different diseases.
- By integrating transcriptomics, proteomics, and other omics data, we developed a ferroptosis-related gene prognostic model for gynecological tumors, which may represent the first such effort in this field
- The elucidation of the complex regulatory network governing ferroptosis in gynecological pathologies holds both theoretical significance and clinical translational promise, prompting this study to systematically integrate existing knowledge within this field.
- The article also emphasizes that in the early stages of ferroptosis research within gynecological diseases, it demonstrates substantial theoretical and clinical potential, especially in the realms of personalized therapy and precision medicine.
- Emphasis on ferroptosis’s role in personalized therapy and precision medicine, particularly through its modulation in high-impact gynecological diseases.

## 1. Introduction

The metal element iron operates as a linchpin in the coordinated sequence constituting ferroptosis, a specialized subset of programmed cell death [1–4]. Before transitioning to mature analytical frameworks, Dixon and colleagues made groundbreaking descriptions of ferroptosis. They established ferroptosis as a novel erastin-dependent mechanism of non-apoptotic cell death fundamentally dependent on iron [5]. This paradigm-defining articulation furnished indispensable theoretical scaffolding for subsequent scholarly exploration.

**Figure 1.**
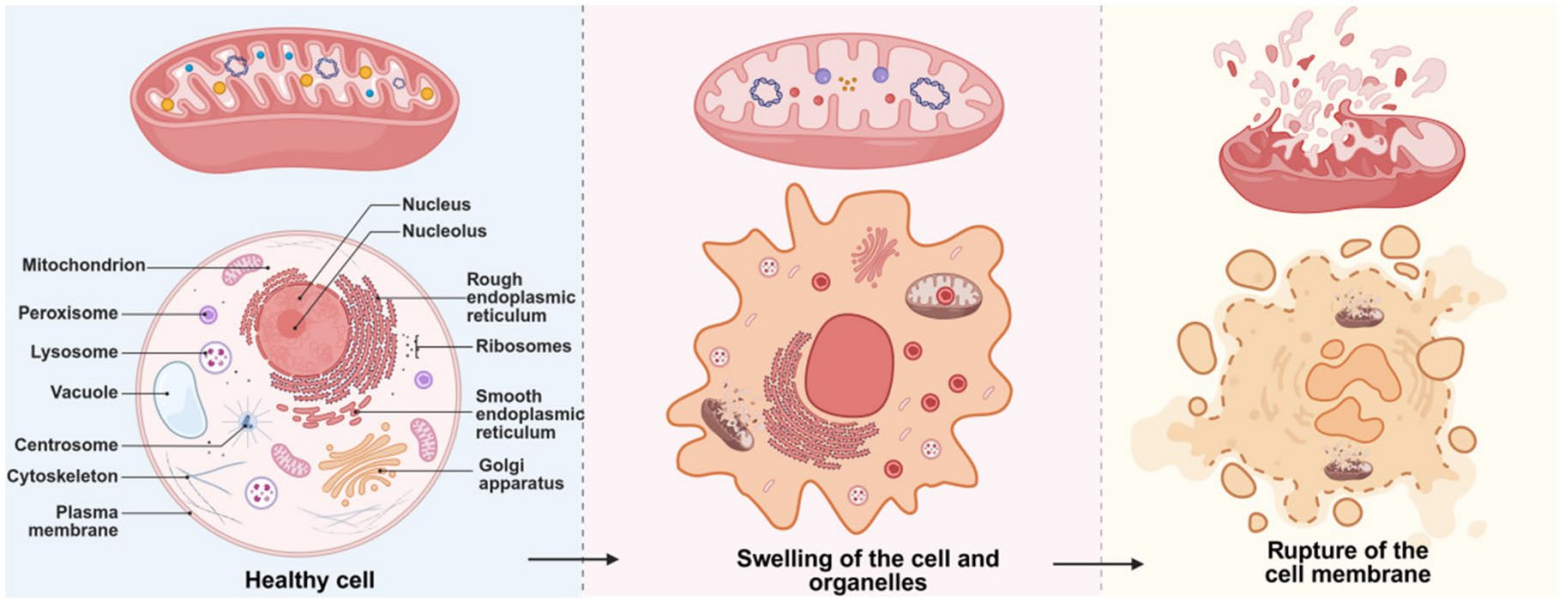
Ferroptotic cell model: Ferroptosis initiates a lipid peroxidation cascade, generating hydroxyl radicals that attack the mitochondrial inner membrane. GSH depletion leads to GPX4 inactivation, resulting in compromised antioxidant capacity that exacerbates membrane damage and oxidative stress. Excessive iron accumulation disrupts mitochondrial electron transport processes, producing ROS such as superoxide anions.

The specific manifestation of this cell death pathway presents multiple sets of morphological, biochemical, and genetic hallmarks. From a morphological perspective, cells undergoing ferroptosis show significant mitochondrial shrinkage. Normally functioning mitochondria gradually decrease in volume and their structure becomes irregular. Notably, the dissolution or loss of mitochondrial cristae is particularly striking. Functionally imperative mitochondrial cristae, products of the inner membrane’s cristae formation, rely on intact architecture; breaches in this barrier exert deleterious impacts on mitochondrial operations. Mitochondria, known as the “powerhouses” of the cell, when impaired, jeopardize the cell’s energy supply. Additionally, the outer mitochondrial membrane damage precipitates structural collapse and functional breakdown, negating mitochondria’s ability to uphold cellular metabolism, thereby posing a serious threat to the survival of the entire cell (Figure 1).

From a biochemical perspective, ferroptosis is characterized by intracellular GSH depletion. As a central antioxidant, GSH neutralizes reactive oxygen species (ROS) and maintains redox balance; its exhaustion during ferroptosis severely weakens cellular oxidative defense. Concurrently, *GPX4* activity declines, directly disabling the clearance of lipid peroxides and triggering lethal oxidative membrane damage—*GPX4* dysfunction thus serves as a core driver of pathological cell and tissue injury in gynecological diseases. *Nicotinamide Adenine Dinucleotide Phosphate (NADPH)*-dependent lipid peroxidation and iron‑dependent ROS production further amplify this process. ROS and lipid peroxides act as pivotal markers that form a self‑reinforcing vicious cycle to sustain ferroptotic progression, shaping cell fate and promoting disease development [1,5,6]. Excessive ROS and lipid peroxidation disrupt membrane structure and integrity, increase permeability, and ultimately induce cell death, which underlies malignant progression, chemoresistance, chronic inflammation, and organ dysfunction in EC, OC, EMS, and PCOS [7,8]. This mechanistic cascade highlights the GSH/*GPX4*/lipid peroxidation axis as a clinically actionable target for predicting disease prognosis, reversing drug resistance, and developing targeted therapies against gynecological diseases [9–11].

At the genetic level, there are also specific regulatory mechanisms related to it. Ferroptosis stands validated in its correlation with multiple human pathologies, notably ischemia-reperfusion injury, degenerative maladies, and carcinogenesis [5,8,12–19]. In obstetric and gynecological science, the fledgling field of ferroptosis research is overshadowed by its profound implication—the unique control it exerts over cell death demands detailed study in disease contexts. Such findings may unlock transformative opportunities in managing complex disease entities through advanced diagnostic tools and targeted interventions, introducing hitherto uncharted solutions. By clarifying defined regulatory roles and biochemical cascades of ferroptosis in obstetric and gynecological diseases, we can not only more accurately understand the patterns of disease development but also develop more targeted and effective treatments, thereby bringing substantial improvements and enhancements to the health and well-being of women.

## 2. Research progress on ferroptosis in gynecological diseases

### 2.1. Research progress on ferroptosis in EC

EC constitutes a leading gynecologic malignancy, with recent decades witnessing sustained increases in case volume and a worrying decline in median age at presentation [20–24]. Proteogenomic research on EC has shown that in early low-grade EC tissue samples, ferroptosis-related pathways are significantly dysregulated [25]. This suggests that abnormalities in ferroptosis may be related to the progression of EC. Amidst evolving scientific exploration, the functional relevance of ferroptosis-linked genetic networks to EC pathobiology has emerged as a focal point of investigation. Accumulating data corroborate their substantial contribution to neoplastic initiation, clonal expansion, tissue invasion, and distant colonization in EC (Table 1). Comparative transcriptomic analyses consistently detect aberrant ferroptosis gene expression patterns segregating EC from adjacent histologically normal tissues, predictive of disease outcome. Systematic characterization documents repressed *Kelch Like ECH Associated Protein 1 (KEAP1)* and *Heme oxygenase 1* (*HMOX1*) in neoplasm biopsies, juxtaposed with augmented *HSBP1, SAT1, CISD1*, and *GPX4* expression in neoplastic versus non-transformed tissue sectors [26]. Recently, the research from Hou et al. establishes a role for long non-coding RNAs (lncRNAs) as architectural scaffolds coordinating *DNA methyltransferase 1* (*DNMT1*), directing its recruitment to the promoter domain of cell *cycle-dependent protein demethylase 1 (CDO1)*. This interaction elevates local DNA methylation density, silencing *CDO1* transcription and consequently dampening ferroptotic activity [27]. Pan et al. established that endometrial stem cells (EnSCs) suppress ferroptosis induced by cisplatin, aiding in cellular recovery. Situated at the heart of this response is *nuclear factor erythroid 2-related factor 2 (NRF2*), whose actions as a key effector are responsible for the observed protection [28]. Shao’s team established that *microsomal GSH S-transferase 1 (MGST1)* governs oxidative stress dynamics, acting as a safeguard against iron-induced cell death in malignant contexts. Overexpression of *MGST1* aligns with cancer progression, worse patient survival, and disrupted immune surveillance, underscoring its potential as a prognosticator for EC [29]. In their study, Zhang and colleagues documented heightened *circular RNA RAPGEF5 (circRAPGEF5)* levels in EC specimens. Functionally, this circRNA collaborates with *RNA binding protein fox-1 homolog 2 (RBFOX2)* to compromise iron pool stability and suppress lipid peroxide generation, thereby establishing ferroptosis resistance and accelerating tumor cell growth. Crucially, experimental manipulation of *RBFOX2* (either silencing or enforced expression) abrogates these oncogenic effects [30]. A novel mechanism involving the *RNA-binding motif protein 3 (RBM3)/solute carrier family 7 member 11 (SLC7A11)* signaling axis was uncovered by Wang’s team, demonstrating how NaBu stimulates ferroptosis to constrain iron-catalyzed cellular demise within neoplastic contexts. The significance of this finding points to the axis’s potential application as a diagnostic tool and targeted therapy for EC [31]. *Adenylyl cyclase-associated protein 1 (CAP)* regulates the lactacylation of histone H3K18 mediated by *Histone deacetylase 3 (HDAC3)*, activates *Tumor Protein P53 (TP53)* transcription, and drives ferroptosis, elucidating a fresh mechanistic basis for CAP in EC treatment and introducing a promising new therapeutic modality [32]. In addition, research emerging findings highlight that high expression of *SOX4, GPX4, SLC11A2, SLC7A11, SAT1, TP53, NRF2, NF-κB, FOXA2*, and *CAPG* in endometrial tissues indicates a better prognosis, while high expression of *GLP1R, ACAT2, LDHA, CDKN2A, FZD7, ACTN4, MYH10, LCN2, hsa-mir-4758*, and *ENPP2* in endometrial tissues indicates a worse prognosis [26,33–42]. Groundbreaking investigations report that optimized drug delivery vehicles amplify chemosensitivity in EC cells, presenting a viable approach for refined therapeutic targeting. Crucially, the safety parameters of these modalities have been validated in controlled *in vitro* environments [43–47]. Natural product-derived agents are emerging as critical tools against cancer. Notably, statins, quercetin, amentoflavone, resveratrol, and fisetin impede EC cell growth through the induction of ferroptosis, validating their translational utility [48–51]. These findings indicate that ferroptosis-inducing strategies may hold significant therapeutic promise in the context of EC, potentially complementing existing treatment modalities.

**Table 1.** Research progress on ferroptosis in EC.

Intervention factors	Samples	Experiments		Effects and mechanisms	References
		*In vivo*	*In vitro*		
LncRNA	Human EC tissues and matched surrounding normal tissues	Xenograft BALB/c nude mice for Ishikawa cells	Human EC cells (Ishikawa, HEC-1-A)	*CDO1*↓, ferroptosis↓	[27]
CircRAPGEF5	Human EC tissues and matched surrounding normal tissues	Xenograft BALB/c nude mice for KLE and Ishikawa cells	Human EC cells (KLE, Ishikawa, HEC-1-A, HEC-1-B, RL95-2)	*RBFOX2*↓, ferroptosis↓	[30]
Sodium butyrate	–	Xenograft BALB/c nude mice for Ishikawa cells	Human EC cells (Ishikawa, HEC-1-B)	*RBM3↑, SLC7A11↓*, ferroptosis↑	[31]
Cold atmospheric plasma	Human EC tissues	Xenograft BALB/c nude mice for HEC-1B cells	Human EC cells (Ishikawa, HEC-1-B)	*HDAC3↓, TP53*↑, ferroptosis↑	[32]
SOX4	–	Xenograft BALB/c nude mice for Ishikawa cells	Human EC cells (AN3-CA, RL95-2, HEC1A, Ishikawa)	*TP53↓, SLC7A11*↑, ferroptosis↓	[33]
GLP1R	Human EC tissues and matched surrounding normal tissues	–	Human EC cells (RL95-2, HEC-1A, KLE, Ishikawa, AN3-CA)	*SLC7A11*↑, ferroptosis↓	[36]
ACAT2	–	–	Human EC cells (HEC- 1-A, HEC-50, Ishikawa)	*FOXA2*↓, ferroptosis↓	[37]
Simvastatin	–	–	Human EC cells (Ishikawa)	*RAS/MAPK↓*, ferroptosis↑	[48]
Quercetin	–	–	Human EC cells (HEC-1-A)	*GPX4*↓, ferroptosis↑	[49]
Amentoflavone	–	–	Human EC cells (KLE)	*ROS/AMPK/mTOR↓,* ferroptosis↑	[50]

### 2.2. Research progress on ferroptosis in EMS

EMS, a prevalent gynecological disorder impacting approximately 10% of females in their childbearing years, manifests as extrauterine implantation of metabolically active endometrium-like tissue. Its pathogenesis is widely attributed to retrograde menstruation phenomena [52–55]. A growing body of work associates perturbed ferroptotic mechanisms with the clinical manifestation of EMS (Table 2) [9,56–60]. Recently, Huang and others utilized bioinformatics methods to discover that *FZD7*, an ferroptosis suppressor gene, is significantly upregulated in EMS lesions and emerging as a plausible therapeutic intervention point for EMS [61]. Earlier research documents a pronounced overexpression of *Fibulin 1 (FBLN1)* in ectopic endometrial stromal cells; this dysregulated *FBLN1* acts to impede *EGF-Like Fibulin Extracellular Matrix Protein 1 (EFEMP1)*-dependent ferroptotic pathways, ultimately bolstering cellular viability and migratory potential [62]. Additionally, in endometrial epithelial stem cells (EESCs), the onset of ferroptosis is accompanied by a pronounced decrease in *Enhancer Of Zeste 2 Polycomb Repressive Complex 2 Subunit (EZH2)* expression, a key transcriptional regulator. Leveraging EZH2 inhibition to drive ferroptotic cell death may constitute an effective treatment modality for ovarian EMS [63]. Contemporary analyses demonstrate that silencing related genes, such as *PDZ and LIM domain 3 (PDLIM3)*, *neuronally expressed developmentally downregulated 4 (NEDD4)*, and *circ_0008927* triggers and hastens the process of cellular ferroptosis, which associates with diminished proliferative, migratory, and invasive capabilities of EMS cells, thereby paving novel therapeutic avenues for EMS management [64–66]. Such findings postulate that ferroptosis constitutes a homeostatic regulatory pathway, with its disruption driving EMS development, propagation, and metastatic spread. Current research positions ferroptosis-linked genetic signatures as predictive indicators of EMS pathogenesis, such as high expression levels of *CD24, FAS, PIK3CA, PTPN11, TGFBR2, CFL1, CHMP6,* and *CISD3* which typically predict an increased likelihood of EMS, while high expression levels of *BECN1, GSK3B, IREB2, TIGIT, GPX4, ACSL4, FADD, FLOT1* and *HLA-DMA* indicate a lower likelihood of EMS [67–70]. Sonia Chadha used the CIBERSORTx tool to demonstrate that *TP53, HMOX1, CAV1, CDKN1A, CD44, EPAS1, SLC2A1, MAP3K5, GCLC,* and *FANCD2* are hub genes in EMS, and found a link between immune dysregulation and ferroptosis in EMS, implicating immune system deregulation and ferroptosis as fundamental drivers in the pathophysiology of EMS [71]. The dynamic crosstalk between lncRNAs and microRNAs as governing elements in ferroptosis represents a key area of investigation. As an illustrative case, Chen’s team defined a regulatory cascade in which *lncRNA ADAMTS9-AS1* commands the *miR-6516-5p/GPX4* pathway to curb ferroptosis in endometrial stromal cells [72]. Inhibiting *lncRNA MALAT1* can promote erastin-induced ferroptosis within endometrial stromal cells, possibly related to the regulation of the *miR-145-5p/mucin 1 (MUC1)* signal [73]. Resveratrol has been shown through recent experimental data to activate the *TP53/SLC7A11* pathway by inhibiting *miR-21-3p*, subsequently eliciting ferroptosis and acting as a protective mechanism against EMS. The observed phenomenon validates resveratrol’s therapeutic potential in harnessing cell death machinery for disease interception [74]. However, Presently, there exists a void in the realm of efficient drug candidates interfacing with ferroptosis regulators that have entered clinical trial stages. Prevailing research supports bioengineered systems optimized for EMS receptor targeting through carrier and ligand modifications. In alignment, Fu et al.’s findings reveal β-ELE induces ferroptosis *via Mitogen-Activated Protein Kinase 1 (MAPK)* and *Signal Transducer And Activator Of Transcription 3 (STAT3)* signaling, markedly decreasing *STAT3* and *MAPK14* phosphorylation [75]. To counteract ferroptosis, *Interleukin 33 (IL-33)* utilizes the *p38/JNK/Activating Transcription Factor 3 (ATF3)/SLC7A11* signalling cascade to boost *SLC7A11* expression in EESCs, a mechanism validated by research from Wu and colleagues. This can alleviate diseases in EMS model mice, suggesting that this “immune-ferroptosis” combination therapy may be a safe and effective new treatment strategy [76,77]. By interacting with the active site of prion protein (PrP), creatine hinders Fe^3+^ reduction to Fe^2+^, curtails iron uptake, forestalls ferroptotic cell death, alleviates oxidative damage and lipid peroxidation, and accelerates EMS development. These findings illuminate EMS disease dynamics, suggesting creatine as a viable therapeutic candidate [78]. When targeting the ferroptosis pathway is combined with biomedical engineering technology, this intervention represents an encouraging therapeutic avenue for addressing EMS [79,80].

**Table 2.** Research progress on ferroptosis in EMS.

Intervention factors	samples	Experiments		Effects and mechanisms	References
		*In vivo*	*In vitro*		
FAC	Human ectopic endometrial tissues	–	EESCs	*EZH2↓*, ferroptosis↑	[63]
PDLIM3	–	–		Gli1↑, Hedgehog↑, ferroptosis↓	[64]
NEDD4	–	–	EESCs	*PTGS2↓*, ferroptosis↓	[65]
circ_0008927	Human ectopic endometrial tissues	–	Primary human endometrial stromal cells (HESC), obtained immortalized endometrial stromal stem cells (hEM15A)	*PROM2↑*, ferroptosis↓	[66]
LncRNA	Human ectopic endometrial tissues	Xenograft BALB/c nude mice of EMS for estradiol valerate	EESCs	*GPX4*↑, ferroptosis↓	[72]
LncRNA MALAT1	Human ectopic endometrial tissues and normal tissues	Xenograft BALB/c nude mice of EMS for 17-β-estradiol-3-benzoate	EESCs, normal endometrial stromal cells (NESCs)	*MUC1*↑, ferroptosis↓	[73]
Resveratrol	Human ectopic endometrial tissues	Xenograft BALB/c nude mice of EMS for estradiol valerate	EESCs, NESCs	*miR-21-3p↓, TP53↑, SLC7A11*↓, ferroptosis↑	[74]
β-ELE	–	Xenograft BALB/c nude mice of EMS for estradiol valerate	HEM12Z	*GPX4↓, p-STAT3↓, p38↓,* ferroptosis↑	[75]
IL-33/ST2	Human ectopic endometrial tissues and normal tissues	Xenograft BALB/c nude mice of EMS for estradiol valerate	EESCs	*ATF3↓, SLC7A11↑, p38/JNK↓,* ferroptosis↓	[76]
Creatine	Human ectopic endometrial tissues and normal tissues	Xenograft BALB/c nude mice of EMS for estradiol valerate	EESCs, NESCs	*PrP*↓, ferroptosis↓	[78]

### 2.3. Research progress on ferroptosis in polycystic ovary syndrome (PCOS)

Characterized by its high incidence among women during their childbearing years, PCOS stands as a quintessential endocrine pathology in gynecological practice, characterized mainly by chronic anovulation and hyperandrogenism [81–87]. As research has progressed, numerous scholars have reported on the detailed mechanisms of ferroptosis in PCOS (Table 3). In experiments conducted by Geronikolou and others, Within the intricate network driving PCOS progression, TP53 emerges as the predominant signalling hub validated by experimental models [88]. A comparative transcriptomic analysis identified a signature of altered ferroptosis regulation in PCOS. Key inhibitory components including GSH, *GPX4, TFR1, FTH1, ATF3, DDIT4, LPIN1, NOS2, NQO1, SLC2A1*, and *SLC2A6* were repressed, while activators like *JUN, STAT3, HMOX1, HMGB1, PPAR-α, BCAP31, EDEM1, TRIB3*, and *ERMP1* were overexpressed. Within this signalling network, ferroptosis emerges as a modulator of disease susceptibility, laying groundwork for systems biology approaches in etiological research [89–95]. Chen and others demonstrated through mouse models that targeting the *sterol regulatory element-binding factor-2 (SREBF2)/arachidonate 15-lipoxygenase (ALOX15)* axis, Period1 (PER1) orchestrates ferroptosis alongside lipid homeostatic imbalance in granulosa cells harvested from diet-induced PCOS murine models, leading to polycystic ovarian morphology. Consistent with this, a profile of high *Arachidonate 15-Lipoxygenase (ALOX15)* and low *Sterol Regulatory Element Binding Transcription Factor 2 (SREBF2)* mRNA levels was observed in the PCOS group [96]. According to research from Peng and coworkers, the therapeutic benefits of metformin on the ovaries of PCOS mice involve the suppression of ferroptosis, which is orchestrated through the *Sirtuin 3 (SIRT3)/AMPK/mTOR* signalling cascade, providing a new supplement to the treatment mechanism [97]. Ni and others also utilized a mouse model to confirm that Egl-9 Family Hypoxia Inducible Factor 1 (EGLN1) induces apoptosis in PCOS mice through the ferroptosis axis, exerting a therapeutic effect on PCOS [98]. The activity of *methyltransferase-like 3 (METTL3)*, which curbs ferroptosis through the *M6A/GPX4* pathway, has been linked to adverse outcomes, specifically the enhancement of ovarian fibrosis and the pathogenesis of PCOS [99]. According to recent findings by Zhou et al. the homeostatic management of ferroptosis in granulosa cells through the *pyruvate dehydrogenase kinase 4 (PDK4)/Janus kinase (JAK)/STAT3* pathway by Rad3-related (ATR) highlights its therapeutic candidacy for PCOS, particularly *via PDK4*-directed intervention [100]. Through modulation of the *AMPK/NRF2* pathway, lycopene (LYC) administration demonstrates therapeutic efficacy in PCOS rats by restoring estrous cycle regularity, improving ovarian morphology, and blocking ferroptosis induction [101]. Exosomes (EXOs) have been increasingly implicated in promoting ferroptosis in granulosa cells (GCs) and precipitating a PCOS-like phenotype. Studies show that while exosomal *miR-128-3p* inhibits ferroptosis, its protective effect is reversed by CSF1 overexpression, primarily through the *p38/JNK/SLC7A11* pathway [102]. The combined treatment with AgNPs and Zileuton demonstrates significant anti-inflammatory, anti-apoptotic, and anti-ferroptotic properties that effectively mitigate the symptoms associated with PCOS [103]. Crucially, the intake of n-3 PUFAs exerts regulatory control over ovarian granulosa cell fate in PCOS, dampening proliferation and stimulating ferroptosis. This therapeutic action is orchestrated through the *Yes1 Associated Transcriptional Regulator (YAP1*)/NRF2 signaling pathway, ultimately leading to improved hormonal profiles [104]. However, some studies have indicated that *NEDD4 Like E3 Ubiquitin Protein Ligase (NEDD4L)* may exacerbate endocrine disorders and reproductive dysfunction in PCOS patients, thereby promoting the development of PCOS [105]. Crucially, the data reveal that ferroptosis acts as a master switch influencing granulosa cell behavior (proliferation and secretion) within the polycystic ovary environment. This makes ferroptosis an attractive target for developing much-needed new treatments for PCOS. For example, baicalin can improve the prognosis of PCOS patients by reducing chronic inflammation and ferroptosis caused by cellular oxidative stress [106]. Quercetin can alleviate PCOS by inhibiting oxidative stress and ferroptosis [107]. In line with Wang et al.’s observations, berberine (BBR) initiates ferroptosis *via* dynamic reconfiguration of the *circ_0097636/miR-186-5p/SIRT3* signalling module, exacerbating oxidative stress burden in PCOS cell cultures, which constitutes a promising therapeutic modality for PCOS intervention [108]. Leonurine (SCM-198) exerts its therapeutic efficacy in PCOS by intervening in ferroptosis pathways within GCs, operating *via* the *SLC7A11/GPX4* axis while harnessing bacterial metabolites to restore ovarian function and alleviate clinical manifestations [109]. The concept of modulating ferroptosis emerges as a compelling therapeutic avenue for PCOS, supported by novel pharmacological evidence. Platycotin D, for example, exerts its protective effects against ovarian damage in PCOS through priming the *CD44/SLC7A11* signalling nexus to limit ferroptosis in GCs [110]. Further supporting this strategy, nuciferine (NF) has been documented as a ferroptosis inhibitor effective against PCOS pathogenesis [111]. Challenging simplifying views, evidence shows that 1,25-dihydroxyvitamin D3 (1,25D3) can orchestrate a specialized form of ferroptosis featuring prominent ROS and lipid peroxidation, which in turn performs a protective role by moderating global oxidative stress [112]. Together, these investigative findings strongly suggest that therapeutic strategies aimed at regulating ferroptosis possess substantial clinical potential for PCOS management.

**Table 3.** Research progress on ferroptosis in PCOS.

Intervention factors	Samples	Experiments		Effects and mechanisms	References
		*In vivo*	*In vitro*		
PPAR-α	–	Mouse PCOS model for DHEA	KGN cell	*FADS2↑,* ferroptosis↓	[91]
–	Blood samples from PCOS	–	–	*FTH1↓, HAMP↓, GPX4↓, A2M↓, HP↓*, *NCOA4* ↑, ferroptosis↑	[94]
PER1	–	Mouse PCOS model for DHEA	KGN cells	*SREBF2/ALOX15↓*, ferroptosis↑	[96]
Metformin	–	Mouse PCOS model for a high-fat diet and letrozole	–	*GPX4↑, SIRT↑, AMPK/mTOR↑*, ferroptosis↓	[97]
Roxadustat	Ovarian GCs, follicular fluids, blood samples from PCOS	Mouse PCOS model for DHEA	–	*HIF-1α↓, EGLN↑, FABP3↓*, ferroptosis↓	[98]
METTL3	–	Mouse PCOS model for DHEA	Primary mouse GCs	*GPX4*↓, ferroptosis↑	[99]
Atractylodin	–	Mouse PCOS model for DHEA	KGN cells	*GPX4↑, PDK4↑, JAK-STAT3↑*, ferroptosis↓	[100]
Lycopene	–	Mouse PCOS model for DHEA	–	*AMPK/NRF2↑*, ferroptosis↓	[101]
Serum exosomes	–	Mouse PCOS model for a high-fat diet and letrozole	Primary mouse GCs, KGN cells	*CSF1↑, p38/JNK↑, SLC7A11↓*, ferroptosis↑	[102]
AgNPs and zileuton	–	Mouse PCOS model for letrozole	–	*HO-1↑, GPX4*↑, ferroptosis↓	[103]
n-3 PUFA	–	Mouse PCOS model for DHEA	KGN cells	*YAP1↓, Hippo*↑, ferroptosis↑	[104]
NEDD4L	–	Mouse PCOS model for DHEA	KGN cells	*GPX4↓*, ferroptosis↑	[105]
Baicalein	–	Mouse PCOS model for DHEA	Primary mouse GCs, KGN cells	*GSH↑, GPX4↑, ACSL4↓*, ferroptosis↓	[106]
Quercetin	–	Mouse PCOS model for DHEA	–	*CHAC1↓, PTGS2↓, ACSL4↓, RGS4↑,* ferroptosis↓	[107]
Berberine	Follicular fluid from PCOS	–	KGN cells	*SIRT3↑, circ_0097636↑, miR-186-5p↓,* ferroptosis↓	[108]
Leonurine	Follicular fluid from PCOS	Mouse PCOS model for DHEA	KGN cells	*GPX4↑, SLC7A11↑,* ferroptosis↓	[109]
Platycodin D	Follicular fluid from PCOS	Mouse PCOS model for DHEA	KGN cells	*CD44↑, SLC7A11↑,* ferroptosis↓	[110]
Nuciferine	–	–	KGN cells	*SOX2↑, GPX4↑, SLC7A11↑,* ferroptosis↓	[111]
1,25-Dihydroxyvitamin D3	–	–	KGN cells	*GPX4↑, SLC7A11↑, ACSL4↓*, ferroptosis↓	[112]

### 2.4. Research progress on ferroptosis in OC

OC is a common gynecological malignancy. The absence of prominent early warning signs coupled with the aggressive metastatic nature of OC contributes to its poor prognosis, undermining women’s health and negatively impacting their life expectancy [113–118]. As a relatively new development, provoking ferroptosis has surfaced as a potentially powerful new method for fighting OC. (Table 4) [119–125]. Emerging evidence supports ferroptosis as a regulatory axis in OC progression. Ferroptosis in OC cells are driven by iron metabolism imbalance and redox dysregulation. Core mechanisms: imbalanced iron import (TFR1/DMT1) and export (FPN), *Nuclear Receptor Coactivator 4 (NCOA4)*-mediated ferritinophagy-released iron, causing lip expansion and ROS burst; mitochondrial iron disorder accelerates ROS, *Dihydroorotate Dehydrogenase (DHODH)* defends; *Acyl-CoA Synthetase Long Chain Family Member 4 (ACSL4)/ Arachidonate 15-Lipoxygenase (ALOX15)* promote lipid peroxidation for ferroptosis execution; hypoxia forms a bidirectional network *via Hypoxia Inducible Factor 1 Subunit Alpha (HIF-1α)* (upregulating TFR1, modulating *GPX4*/ROS), affecting the “iron-addicted” phenotype and chemoresistance [126,127]. Bioinformatic analyses have enabled researchers to develop ferroptosis-based prognostic signatures for OC patients [128–132]. Key predictors include *SLC7A11, SOX2, FH, GCH1, MYCN, FURIN, SQLE, PARK7, HOXB3*, and *PVR*—whose elevated expression correlates with poor prognosis—while high levels of *ALOX12, TIGIT, STUB1, LAG3, CTLA4, IDO1, ICOS, CD27* and *IL2RB* indicate favorable outcomes. Mechanistically, the *junctional adhesion molecule 3 (JAM3)* induces heightened cisplatin resistance and cellular adhesion in OC cells concomitant with suppression of the *NRF2*/*ferroptosis suppressor protein 1 (FSP1)*-mediated ferroptotic program, linking its activity to adverse prognosis [133]. Furthermore, the tumor suppressor *BRCA1* exerts its anti-tumoral effects by promoting *GPX4* multi-ubiquitination and degradation, thereby sensitizing cancer cells to ferroptosis [134]. Inspiring progress in ferroptosis-targeted OC therapy underscores its translational potential. The Jiang et al. study shatters ferroptosis resistance by thwarting *ACSL4* exocytosis *via VIPAS39* inhibition [135]. By elucidating the *Serine-protein kinase (ATM)/AMPK/solute carrier family 2 member 3 (SLC2A3)* axis as a master regulator of *oxidative stress-induced growth inhibitor 1 (OSGIN1)*-dependent ferroptosis, Deng et al. establish a strong rationale for its development as a clinical strategy [136]. Crowning these insights, sodium butyrate (SB) validates the *Rho GTPase Activating Protein 10 (ARHGAP10)/GPX4* axis as a potent ferroptosis inducer, elevating ROS and annihilating cancer cell viability – a triumph that paves the way for revolutionary anticancer treatments [137]. Ferroptosis resistance in OC arises from multiple regulatory networks. For instance, Han et al. identified *six-transmembrane epithelial antigen of prostate 3 (STEAP3)* as a fundamental deterrent, highly expressed in OC where it suppresses ferroptosis regulated by the *TP53/SLC7A11* conduit, marking it as a biomarker for poor prognosis [138]. In contrast, the lncRNA *TPT1 Antisense RNA 1 (TPT1-AS1)* actively blocks erastin-triggered ferroptosis by regulating the *GPX4/ (KH RNA Binding Domain Containing, Signal Transduction Associated 3) KHDRBS3* interaction, promoting tumor survival [139]. Compelling evidence for the central role of *GPX4* comes from the discovery that *paired Box 8 (PAX8)* acts as a *GPX4*-dependent susceptibility gene; targeting *PAX8* alongside *RAS-selective lethal 3 (RSL3)* successfully induces ferroptosis and halts tumor growth *in vivo* [140]. Compounding these challenges, serum/*glucocorticoid regulated kinase 1 (SGK1)* in the *phosphoinositide 3-kinase (PI3K)* pathway provides dual protection against ferroptosis in high-grade serous OC – *via* an *NRF2*-dependent arm enhancing antioxidant capacity and an *NRF2*-independent arm fostering protective adipogenesis through *mTOR/Sterol Regulatory Element Binding Transcription Factor 1 (SREBP1)/Stearoyl-CoA Desaturase (SCD1)* [141]. The convergence of ferroptosis induction and traditional antitumor therapy presents a promising strategy, delivering enhanced therapeutic outcomes and overcoming prevalent drug resistance. Illustratively, elevating *DnaJ Heat Shock Protein Family (Hsp40) Member C15 (DNAJC15)* expression drives ferroptosis in OC cells, boosting lipid peroxidation and restoring their sensitivity to cisplatin [142]. Mechanistically, the *mTOR/eIF4E binding protein 1 (4EBP1)* axis plays a key role by repressing *SLC7A11* synthesis, creating a window for MEK inhibitors to reactivate ferroptosis in resistant populations [143]. Supporting this, Wu and colleagues demonstrated that metformin, particularly under glucose-limited conditions, powerfully enhances apoptosis and ferroptosis in OC cells through focalizing interventions on the *Nicotinamide adenine dinucleotide Ubiquinone Oxidoreductase Subunit B4 (NDUFB4)* moiety of mitochondrial complex I [144]. Moreover, metformin’s ferroptosis-promoting effects involve upregulating *RNA Binding Motif Single Stranded Interacting Protein 3 (RBMS3)*, leading to increased Monodehydroascorbate reductase (MDA) and Fe^2+^, reduced GSH, and suppressed tumor cell growth and spread [145]. Completing this suite of evidence, niraparib effectively induces regression in OC peritoneal metastases through *CD36*-mediated ferroptosis driven by elevated lipid oxidative damage. Additionally, literature documents progesterone promotion of ferroptosis and causes mitochondrial damage by upregulating palmitoleic acid, thereby enhancing the activity of niraparib in OC and prolonging patient survival [146,147]. Emerging strategies increasingly focus on modulating ferroptosis to enhance OC therapy. Pharmacologically, *Sphingosine Kinase 1 (SPHK1)* inhibitors demonstrate this principle by acting upon the *NF-κB* pathway to curtail *NRF2* transcription, thereby sensitizing OC cells to olaparib-induced ferroptosis [148]. Similarly, sodium citrate induces ferroptosis by disrupting the *Ca^2+^/Calmodulin Dependent Protein Kinase Kinase 2 (CAMKK2)/AKT/mTOR* signaling cascade, which lowers cytoplasmic Ca^2+^ levels and consequently elevates mitochondrial ROS, boosting the efficacy of conventional chemotherapeutics [149]. Another targeted approach involves fludarabine, which impedes OC progression by inhibiting the *N-acetyltransferase 10 (NAT10)/Acyl-CoA thioesterase 7 (ACOT7)* axis to promote ferroptosis [150]. The potential of natural compounds in this realm is particularly promising. Different classes of small molecules have been substantiated as efficient promoters of ferroptosis in OC cells. Artemether, for instance, inhibits the *Homeobox C11 (HOXC11)/Prominin 2 (PROM2)/PI3K/AKT* axis, thereby activating both apoptotic and ferroptotic pathways. In parallel, obunone exerts regulatory control over the *AKT/TP53* axis, characterized by decreased *AKT* phosphorylation concomitant with augmented *TP53* expression, ultimately promoting ferroptosis. Moreover, honokiol establishes itself as a novel ferroptosis activator in OC through direct *OTU Deubiquitinase, Ubiquitin Aldehyde Binding 2 (OTUB2)* engagement and *YAP* signaling repression [151–157]. The cumulative evidence firmly positions ferroptosis induction as a promising approach to retard OC development.

**Table 4.** Research progress on ferroptosis in OC.

Intervention factors	Samples	Experiments		Effects and mechanisms	References
		*In vivo*	*In vitro*		
Angelica sinensis polysaccharide, Cisplatin (DDP)	–	Xenograft BALB/c nude mice for SKOV3/DDP cells	SKOV3, A2780, DDP	*GPX4*↓, ferroptosis↑	[120]
Hermine	–	Xenograft SCID mice for SKOV3 cells	IOSE80, ES2, SKOV3, A2780	*SLC7A11↓, NRF2↓, GPX4*↓, ferroptosis↑	[123]
EC359	Excised cancer tissues	Xenograft SCID mice for ES2 and ID8 cells	OVCAR5, OVCAR8, OVSAHO, OVCAR3, ES2, SKOV3, TOV21G, TOV112D, OV90, COV644, HEK-293T, ID8agg	*LIF/LIFR* autocrine signaling↓, *STAT3*↓, *SLC7A11↓, GPX4*↓, ferroptosis↑	[125]
Squalene monooxygenase	–	Xenograft BALB/c nude mice for SKOV3 cells	SKOV3, ES2, OVCAR3, A2780, HO8910	*GPX4↑, COX2↑, NOX1↑, ACSL4↑,* ferroptosis↓	[129]
STUB1	OC patient tissues	Xenograft BALB/c nude mice for SKOV3/R cells	SKOV3, A2780, PTX resistant cells (SKOV3/R, A2780/R)	*GPX4↓, HOXB3↓, PARK7*↓, ferroptosis↑	[130]
JAM3	–	Xenograft athymic nude mice for SKOV3 cells	SKOV3, OVCAR3	*NRF2/FSP1*↑, ferroptosis↓	[133]
BCAR1	OC tissues	Xenograft BALB/c athymic nude mice for A2780/BRCA1-KO cells	UWB1.289, UWB1.289-BRCA1, A2780, OVCAR3, SKOV3, HCC1937	*GPX4↓,* ferroptosis↑	[134]
VIPAS39	–	Xenograft BALB/c nude mice for CAOV3 cells and patient-derived xenograft (PDX) model	A2780, CAOV3, SKOV3, OVCAR3, OVCAR8	*ACSL4↓*, ferroptosis↓	[135]
OSGIN1	Human ovarian tumor specimens and normal ovarian tissues	Xenograft NOD/SCID nude mice for OVCAR3 cells	SKOV3, ES2, A2780, COC1, OVCAR3	*AMPK↓, SLC2A3↓*, ferroptosis↑	[136]
ARHGAP10, Sodium Butyrate	–	Xenograft BALB/c athymic nude mice for A2780 cells	OVCAR3, A2780	*GPX4↓, PTGS2↓*, ferroptosis↑	[137]
STEAP3	–	Xenograft BALB/c athymic nude mice for SKOV3 cells	SKOV3, A2780 and their non-malignant counterpart (HOSE)	*STEAP3↓, TP53↑, GPX4↓, SLC7A11↓,* ferroptosis↑	[138]
SGK1	–	Xenograft BALB/c athymic nude mice for OVCAR3 cells	OVCAR3, COV362, OVCAR4, SNU119, Kuramochi cells, OVSAHO, PEO1, OVCAR8 and HEK293T	*NRF2/SLC7A11↑, mTOR/SREBP1/SCD1↑,* ferroptosis↓	[141]
cisplatin	–	–	A2780, SKOV3, OC314	*DNAJC15*↑, ferroptosis↑	[142]
MEK inhibitors	–	Xenograft BALB/c athymic nude mice for SKOV3 cells, Xenograft NOD/SCID nude mice for PDX model	A2780, OV90, TOV112D, OVCAR5, OVCAR3, OVCAR4, SKOV3, COV504, OVCAR5R, A2780R, PDCs	*4EBP1↓, SLC7A11*↓, ferroptosis↑	[143]
metformin	–	Xenograft BALB/c athymic nude mice for SKOV3 cells	SKOV3, A2780	*NDUFB4*↑, mitochondrial complex I*↑,* ATP*↓*, ferroptosis↓	[144]
metformin	–	Xenograft BALB/c athymic nude mice for RBMS3	OVCAR8, SKOV3, Hey, A2780 and normal ovarian surface epithelium (HOSEpiC)	*RBMS3↑, MDA↑, Fe^2+^*↑, GSH↓, ferroptosis↓	[145]
Niraparib	–	Xenograft BALB/c athymic nude mice for ID8 cells and PDX model, Xenograft C57BL/6 mice nude for TP53-deficient ID8 cells	OVCAR8, SKOV3, and mouse OC cell line ID8, A2780, OV90	lipid peroxidation↑, *CD36*↑, ferroptosis↑	[146]
Progesterone	–	Xenograft C57BL/6 and BALB/c nude mice for OVCAR3 cells	HGSC, PEO1, BRCA, SKOV3, A2780	Palmitoleic acid↑, ferroptosis↑	[147]
SPHK1	–	Xenograft female athymic BALB/c nude mice for OVCAR8 cells	SKOV3, OVCAR8	*SPHK1↑, NF-κB↑, p65↑, NRF2↑*, ferroptosis↓	[148]
Sodium citrate	–	Xenograft female BALB/c nude mice for SKOV3 cells.	SKOV3, A2780, HOSEpiC, IOSE-80, human embryonic kidney cells (HEK293T)	*CAMKK2/AMPK↓, MCU↓*, mitochondria Ca^2+^↓, ROS↑, ferroptosis↑	[149]
Fludarabine	–	Xenograft BALB/c athymic nude mice for OVCAR3 cells	The HEK293T, SKOV3, OVCAR3	*NAT10↓, ACOT7↓,* ferroptosis↑	[150]

### 2.5. Research progress on ferroptosis in cervical cancer

Cervical cancer (CC), a malignancy originating in the cervix, benefits greatly from early detection programs and heightened public awareness, allowing for surgical intervention at a pre-invasive stage in many cases. Despite these advances, a significant subset of patients presents with advanced-stage disease, for whom systemic drug therapy remains a critical area of investigation [158–163]. Promising new research highlights the clinical relevance of ferroptosis, suggesting that genes associated with this form of cell death may serve as robust diagnostic and prognostic biomarkers for CC (Table 5) [164]. For example, *ANO6, KDM3A, ANGPTL4, P4HA1, PGK1, VEGFA, TFRC, CNIH4, GPX4, SLC7A11, SLC2A14*, and *SQLE* indicate a relatively poor prognosis, while *SLC7A5, TAZ, SOX2, KLF14, MPC1, FBXW7, G6PD, TP53* and *ZNF419* indicate a better prognosis [164–179]. Investigative work linking ferroptosis dysfunction to CC advancement has gained substantial traction. Illustratively, Gong et al. provide strong evidence that *miR-30c-5p* is a potent promoter of ferroptosis in CC cells, a conclusion validated through rigorous gain- and loss-of-function studies showing its antagonistic effect on the *METTL3/kirsten rats sarcoma viral oncogene (KRAS)* module, thereby inhibiting their proliferation and migration and providing new insights [180]. *METTL3* mediates the occurrence of ferroptosis by influencing *COTE-1*, thus inhibiting the progress of CC [181]. Recent advancements have illuminated multiple facets of ferroptosis in CC pathogenesis. For instance, *Methyltransferase 14 (METTL14)* has been shown to enhance sorafenib-induced cell death through utilization of the *FTH1*-potentiated *PI3K/AKT* signaling machinery, thereby impeding CC progression [182]. Offering a counterpoint, the *MALAT1/MiR-182-5p/EPAS1* mRNA pathway takes charge of *EPAS1* regulation. In the context of CC, *EPAS1* expression is prominently elevated, empowering cancer cells to proliferate, invade, and survive by evading apoptosis [183]. The narrative grows more complex with the identification of *mitochondrial carrier 1 (MTCH1)* as the pivotal agent within the program of *mitochondrial-mediated ferroptosis (MMF)*. By targeting the *Forkhead Box O1 (FOXO1)/GPX4* axis, *MTCH1* upregulates the ferroptotic process to inhibit tumor growth in mice, an effect synergistically enhanced when combined with sorafenib [184]. Building upon this, Gboxin orchestrates ferroptosis in CC cells through the coordinated action of the *p62/KEAP1/NRF2* axis. This process severely compromises the cellular antioxidant defenses, pushing the cells toward apoptosis [185]. Such a mechanism highlights ferroptosis as a promising therapeutic target, an approach already being explored with compounds like Erastin and *centromere protein F (CENPF)*. These agents cleverly hijack the *NRF2/HO-1* pathway, causing destructive ROS accumulation and bringing cell proliferation to a halt [186,187]. Yet, intrinsic and extrinsic factors create barriers to this approach. Tumor hypoxia, for one, by bolstering the antioxidant defenses of CC cells, rendering them resistant to ferroptosis and supporting tumor development [188]. Furthermore, host factors within the tumor niche actively block ferroptosis execution. Immunosuppressive *tumor-associated macrophages (TAMs)*, for instance, act to protect CC cells by downregulating *ALOX15* expression, thus blunting a key pathway for cell death [189]. In addition, *lymphoid-specific helicase (HELLS)* has also been proven to promote cancer proliferation by suppressing the expression of NRF2 [190]. Central to their findings, Liao et al. identified a mechanism whereby *Ubiquitin Specific Peptidase 34 (USP34)* prevents ferroptosis; namely, through the effective suppression of the *Cyclic GMP-AMP Synthase (cGAS)/ Stimulator Of Interferon Response CGAMP Interactor (STING)* biochemical axis, upregulates *PIN1* expression and SUMOylation, and thereby promotes the progression of CC [191]. Manipulating ferroptosis stands as a pivotal strategy in the battle against cervical cancer (CC). Crucially, high-risk human papillomavirus (HPV) subverts this process; its E6 and E7 oncoproteins fortify a defensive shield around cancer cells by demethylating the promoter of *Tubulin Alpha 3f Pseudogene (TUBORF) via* the *CREB-binding protein (CBP)/E1A*-binding protein p300 (p300) signaling pathway, actively suppressing ferroptosis and ensuring unchecked proliferation [192]. Challengers to this viral dominance have emerged in the form of targeted pharmaceuticals. Matrine breaches this defense by activating Piezo1, collapsing the antioxidant system and unleashing devastating ferroptotic cell death [193]. Triptolide (Tri) launches a direct assault on the master regulator *NRF2*, dismantling a key survival circuit and sensitizing CC cells to ferroptosis [194]. Furthermore, Chrysotoxine reprogrammes cellular metabolism through the *Putative histidine kinase (PISK)/AKT/MTOR* pathway, turning it against the cancer cell itself *via* ferroptosis [195]. These groundbreaking studies firmly establish ferroptosis induction as a powerful new paradigm for CC therapy. In addition, some newly discovered natural products such as Dihydroartemisinin (DHA), which have low toxicity and few side effects, also show good efficacy in the treatment of CC [196]. Furthermore, Lactobacillus has also been proven to have a significant effect in the treatment of CC [197]. Environmental pollutant 4-nonylphenol (4-NP) combats CC by inhibiting the *MAPK* pathway, a mechanism that reduces antioxidant capacity and induces ferroptosis [198]. This indicates that targeting the ferroptosis-related pathways may solve the major problem of clinical drug resistance in CC.

**Table 5.** Research progress on ferroptosis in CC.

Intervention factors	Samples	Experiments		Effects and mechanisms	References
		*In vivo*	*In vitro*		
miR-30c-5p	–	Xenograft BALB/c athymic nude mice for HeLa cells	CaSki, HeLa, SiHa, HcerEpic	*METTL3↑, m6A↓,* *KRAS↓*, ferroptosis↑	[180]
METTL3	Human cervical cancer specimens and paracancerous tissues	Xenograft BALB/c athymic nude mice for HeLa cells	HeLa, C33A	*METTL3↑, COTE-1↑*, ferroptosis↑	[181]
Sorafenib	Human cervical cancer specimens and paracancerous tissues	Xenograft BALB/c athymic nude mice for HCC9 cells	ME180, HCC9, MS751, SiHa, HeLa, CaSki	*METTL3↑, FTH1↓*, ferroptosis↑	[182]
MTCH1	–	Xenograft BALB/c athymic nude mice for HeLa cells	HeLa, SiHa	*MTCH1↓, FOXO1↓, GPX4↓*, ferroptosis↑	[184]
Gboxin	–	Xenograft BALB/c athymic nude mice for HeLa cells	HeLa, SiHa, HEK-293T	*p62↓, KEAP1↓, NRF2↓,* ferroptosis↑	[185]
hypoxia	–	–	SiHa, HeLa	*SLC7A11↑, GPX4↑,* ferroptosis↓	[188]
TAMs	Human cervical cancer specimens and paracancerous tissues	Xenograft BALB/c athymic nude mice for HeLa cells	HeLa, SiHa, THP-1	*ALOX15↑,* ferroptosis↑	[189]
USP34	–	Xenograft BALB/c athymic nude mice for HeLa cells	HeLa, SiHa	*USP34↑, PIN1↑, cGAS-STING↓*, ferroptosis↓	[191]
HPV	Human cervical cancer specimens and paracancerous tissues	Xenograft BALB/c athymic nude mice for SiHa cells.	C-33A, HeLa, HT-3, SiHa, UPCI-SCC-154, HEK-293T	*E6/E7↑, TUBORF↑, CBP/p300*↑, ferroptosis↓	[192]
Matrine	–	Xenograft BALB/c athymic nude mice for SiHa cells.	SiHa	*Piezo1↑, GPX4*↓, ROS↑, ferroptosis↑	[193]
Tri	–	Xenograft BALB/c athymic nude mice for HeLa cells	HeLa, SiHa	*NRF2/GPX4/xCT* axis↓, ferroptosis↑	[195]
Chrysotoxine	–	–	HeLa	*TP53/GPX4/SLC7A11↓*, ferroptosis↑	[197]
4-NP	–	Xenograft T-cell deficient nude mice engrafted with HcerEpic	HcerEpic.	*MT2A↑, MAPK*↓, ferroptosis↑	[198]

The study of ferroptosis in gynecological diseases reveals both shared and distinct mechanisms. Common pathways include iron dysregulation, lipid peroxidation, and impaired antioxidant defenses (e.g. *GPX4* dysfunction). Disease-specific features emerge: *ACSL4/LPA* promote ferroptosis resistance in EC, while *FZD7* suppresses it in EMS [61,135,152]. PCOS and OC exhibit unique signaling axis differences (*PER1/SREBF2/ALOX15* vs. *MTCH1/FOXO1/GPX4*) [96,184]. Different diseases exhibit distinct phenotypes through specific regulatory pathways. Ferroptosis is inhibited in EC and OC, thereby promoting tumor cell survival and chemotherapy resistance. In EMS and PCOS, aberrant ferroptosis directly contributes to disease development and progression. Therefore, ferroptosis represents a conserved program fine-tuned in a tissue-specific manner, supporting stratified targeted therapy rather than universal strategies and facilitating reliable biomarker identification and clinical translation.

## 3. Research progress on ferroptosis regulators

### 3.1. Research progress on ferroptosis inducers

Pharmacophores eliciting ferroptotic responses operate *via* divergent biological mechanisms, broadly comprising regulators of iron metabolism, antagonists targeting the System xc⁻ transporter, and compounds disrupting *GPX4* enzymatic activity.

#### 3.1.1. Iron metabolism inducer

As an iron-mediated mode of cellular demise, ferroptosis is intrinsically linked to iron regulation [199–203]. Its dependence on iron manifests in two key ways: first, labile iron catalyzes the Fenton reaction, generating abundant hydroxyl radicals that drive non-enzymatic lipid autoxidation; second, the pro-oxidant activity of lipoxygenase enzymes requires iron to sustain their function [6,204–208]. Consequently, artemisinin emerges as a potent neuroprotective agent against ferroptosis. It acts by activating the *KEAP1/NRF2* pathway, restoring neuronal health, depleting GSH, and suppressing lipid peroxidation – a mechanism beneficial in combatting neurodegenerative conditions like Alzheimer’s disease [176,209,210]. Tellingly, a growing body of evidence indicates that artemisinin and its derivatives can effectively modulate ferroptosis in tumor cells [211–213]. For example, Artemisinin (ART) and artemisinin derivatives (ARTEs) can affect the metabolic processes of cancer cells through various pathways. They can interfere with the energy metabolism of cancer cells and reduce ATP production by inhibiting mitochondrial function, thereby weakening the proliferative capacity of the tumor spheroids. In addition, ARTEs exert their effects partly by activating the oxidative stress response in cancer cells, subsequently boosting intracellular ROS production. Consequently, this oxidative stress compromises the integrity of cancer cell DNA and organelles, setting a course for cell death [214]. Currently, quercetin has also been discovered as a potential agent for preventing preeclampsia (PE). Prophylactic supplementation with low-dose quercetin can rescue endothelial dysfunction in mice. Beyond this, targeting the *epidermal growth factor receptor (EGFR)* offers a mechanism to reduce occurrences of selective uterine-placental perfusion reduction, as demonstrated in mouse experiments [215]. Recent years have witnessed mounting evidence from studies indicating that natural products such as resveratrol, curcumin, and baicalein can exert antitumor effects by modulating ferroptosis through multiple targets. Their mechanisms involve iron metabolism remodeling, regulation of the *GPX4*/GSH antioxidant system, modulation of lipid peroxidation, and intervention in ferritin autophagy, yielding novel knowledge that informs the design of cancer treatments with a superior benefit-to-toxicity profile. These agents particularly show potential advantages in overcoming drug resistance associated with conventional chemotherapy [216–222]. Remarkably, researchers discovered that *carnitine palmitoyl transferase 1 A (CPT1A)* acts as a potent guardian against ferroptosis in lung cancer cells. Its strategy involves bolstering antioxidant defenses *via NRF2/GPX4* and throttling back polyunsaturated phospholipid supply by suppressing *ACSL4*. Far from being purely detrimental, this survival mechanism powerfully synergizes with immunotherapy [223]. *Ubiquitin-specific protease 7 (USP7)* regulates ferroptosis by deubiquitinating *stearoyl-CoA desaturase (SCD)*, achieving the effect of treating gastric cancer [224]. Whereas *Fatty Acid Desaturase 2 (FADS2)* usually promotes tumorigenesis, its downregulation paradoxically initiates ferroptosis by lowering the levels of key antioxidative mediators such as GSH, *SLC7A11* and *GPX4*. Consequently, this induced ferroptosis heightens cancer cell susceptibility to oxaliplatin and impedes tumor growth [225]. In recent years, scientists have gradually developed new types of ferroptosis nano-drugs and shown good effects in inducing ferroptosis. Currently, new research indicates that hybrid nanocomposites MnFe2O4/ART/salinomycin (Sali) NPs formed by combining ART, Sali and MnFe_2_O_4_ can significantly increase intracellular Fe^2+^ content and produce more ROS, enhancing their killing effect and showing good antitumor effects, charting a new course for the rational design of superior ferroptosis-inducing agents [226]. The compelling nature of this evidence is reinforced by consistent results across laboratory models and animal models, showing that the specialized micelles (comprising ART, Fe_3_O_4_, and Tween 80) effectively block tumor growth with an inhibition efficiency of up to 85% [227]. Another study found that a core-shell nanoparticle composed of copper (Cu) and erastin (Er) can synergize copper death and ferroptosis, enhancing lipid peroxidation and inducing a strong immune response to promote tumor cell death [228–233]. In addition, several studies have reported that simultaneously loading Fe_3_O_4_, ZnO, and other agents or drugs such as curcumin, cisplatin, or drugs and lactoferrin in nanomaterials can achieve “killing two birds with one stone” and enhance the ability to induce ferroptosis [234–239]. Despite advances, several currently available ferroptosis agents face challenges including poor bioavailability and off-target effects, their clinical prospects are somewhat hindered. However, the development of new ferroptosis nano-drugs is expected to solve these problems.

#### 3.1.2. System xc- inhibitor

The System xc⁻ transporter, comprising *solute carrier family 3 member 2 (SLC3A2)* and *SLC7A11* subunits, is assembled anchored to the plasma membrane. Here, *SLC7A11* serves as the critical effector, facilitating cystine influx essential for GSH biosynthesis [240–244]. Hence, curbing *SLC7A11* expression proves effective in triggering ferroptosis. The realm of System xc- inhibitors features prominently erastin and its derivatives, such as sorafenib, sulfasalazine, glutamate, etc. Dolma et al.’s 2003 research yielded a small molecule exhibiting remarkable selectivity in eradicating engineered tumor cells and named it erastin [5]. In cells dying induced by erastin, no classic features of apoptosis were found, and this death pathway showed no susceptibility to pharmacological blockade by apoptotic inhibitors [245,246]. Treatment with erastin initiates a cascade beginning with the direct inhibition of System xc-. This restricts cystine availability, causing a significant drop in GSH synthesis. Failing to curb ROS effectively, low *GPX4* levels in tumor cells predispose cellular systems to iron-fueled L-ROS amplification, setting off the chain reaction that breaks down polyunsaturated fatty acids [247–250]. Currently, Pu et al.’s study shows that targeting *protein arginine methyltransferase 4 (PRMT4)* may be a potential strategy against nasopharyngeal carcinoma, because the enzyme *PRMT4* provides a defensive function against erastin-initiated ferroptosis in cisplatin-resistant CNE cells, accomplishing this through transcriptional activation of *GPX4* by *NRF2* [251]. Acting upon this premise, Li et al. built bovine serum albumin-stabilized selenium nanoparticles (BSA-SeNPs) functioning as a potent *NRF2* activator. This maneuver orchestrated elevated *GPX4* expression across mRNA and protein compartments, effectively restraining ROS production during erastin-inflicted ferroptosis. This work opens new avenues for developing anti-ferroptosis treatments [252]. Supporting this, recent research has shown that *Early Growth Response 1 (EGR1)* activates the *NRF2/HMOX1* pathway in breast cancer, not only curbing proliferation but also sensitizing cells to erastin-triggered ferroptosis [253]. Conversely, *Golgi Phosphoprotein 3 (GOLPH3)* overexpression in colorectal cancer promotes cell survival by diminishing susceptibility to erastin-induced ferroptosis, identifying it as a promising molecular target to overcome treatment resistance [254]. Lei et al.’s research demonstrates that *BRCA1* deficiency negates *Voltage Dependent Anion Channel 3 (VDAC3)*-mediated resistance to erastin-induced ferroptosis, thereby inhibiting *GPX4* and rendering cancer cells susceptible to ferroptosis. This study not only elucidates the cascade relationship of the *BRCA1/VDAC3/GPX4* pathway in ferroptosis regulation but also pinpoints a potential vulnerability for therapy in *BRCA1*-mutant colorectal cancer [255]. As a result, diminished *SLC7A11* function leads to both the halted proliferation and the deliberate execution of ferroptosis in multiple myeloma cells, while long noncoding RNA (lncRNA) is identified as a key regulatory factor. In addition, by activating *TP53*, erastin consequently enhances the rate of ferroptosis. This process is because erastin can induce tumor cells to produce ROS, activate *TP53*, and then act on downstream pathways. Notably, when *TP53* is activated, it triggers an increase in ROS, serving to intensify erastin’s pro-ferroptotic effects in tumor cells [256–260]. Recent studies have demonstrated that erastin analogs exhibit promising efficacy in inducing ferroptosis and suppressing ferroptosis-related diseases. With relatively stable water solubility and metabolic stability, these compounds demonstrate potent effects, notably their significant capacity to set in motion ferroptosis in tumor cells [261–264]. According to recent evidence, sorafenib possesses ferroptosis-inducing properties. It achieves this effect primarily by blocking the System xc– transporter, thereby restricting cystine entry into cells. It precipitates a state of ER stress, drastically consumes GSH, and underpins the iron-fueled elevation of lipid-derived ROS. At present, a variety of artificially synthesized small molecule inhibitors have shown potential in research. These inhibitors can specifically act on key proteins or enzymes in the ferroptosis signaling pathway, block the endoplasmic reticulum stress and lipid ROS accumulation caused by sorafenib, and offers a clear path forward for translating our understanding of ferroptosis into revolutionary new treatments [182,265,266]. In addition, sulfasalazine can also increase the cell’s ability to take up cystine by acting on the *PI3K/AKT/ERK1/2* pathway and the *TP53/SLC7A11* pathway, maintain the homeostasis of intracellular GSH, and thus enhance the ferroptosis of rheumatoid arthritis. Forefront research has recently delineated the contributions of ferroptosis to a broad array of clinical and biological conditions. First, central to sulfasalazine’s efficacy in rheumatoid arthritis is a complex regulatory network mediating its effects [267]. Second, in a seminal study, Wu et al. utilized a murine model to show that BBR triggers ferroptosis in nasopharyngeal carcinoma cells, primarily through the System Xc-/GSH/*GPX4* axis [268]. They also found that restoring *GPX4* expression could rescue cells from this form of death, underscoring its importance in BBR’s ability to curb cancer spread. Third, through a direct molecular assault on the *KEAP1*, mangiferin successfully blocks ferroptosis, according to dual *in vivo* and *in vitro* evidence. This liberates the *NRF2* transcription factor, setting off a protective cascade through *SLC7A11* and *GPX4* that champions bone formation against osteoporosis [269]. Fourth, icariin has been shown to reduce ferroptosis in chondrocytes, preventing cartilage degradation *via* the *SLC7A11/GPX4* axis. These distinct studies consistently identify the *SLC7A11/GPX4* pathway as a critical lever for controlling ferroptosis [270]. Although System xc- inhibitors have shown good effects in inhibiting diseases, there are no inhibitors of this type applied in the field of gynecological diseases.

#### 3.1.3. *GPX4* inhibitor

*GPX4* falls under the umbrella of the glutathione peroxidase (GPx) family and exists as a dimeric enzyme consisting of two protein subunits, each containing about 205 amino acids. Its molecular weight is about 19 kDa, composed of about 170 amino acids. In mammals, there are multiple isoforms of the enzyme, which are fundamental to sustaining cellular redox balance and fortifying cells to withstand damage from oxidative stress [271–274]. In 2014, research on targeted metabolomics revealed that both enhancing and suppressing *GPX4* expression effectively mitigated the lethal effects of 12 distinct ferroptosis inducers on cells, characterizing *GPX4* as a principal regulator of ferroptosis [275]. Compounds targeting *GPX4*—containing *RAS-selective lethal 3 (RSL3)*, *Fertility inhibition protein (FINO2)*, and *ML162*—directly or indirectly impede *GPX4* function, triggering ferroptosis characterized by elevated ROS production and intracellular peroxide accumulation [276–281]. Concurrently, endogenous metabolic enzymes contribute to ferroptosis regulation. Notably, cysteine/cystine-metabolizing enzymes deplete serum cysteine/cystine pools, limiting GSH biosynthesis substrates and thereby suppressing *GPX4* activity to induce ferroptosis [282–285]. Statins like simvastatin and fluvastatin similarly promote tumor cell ferroptosis through transcriptional repression of *GPX4* [286–289]. Natural compounds exhibit potent ferroptosis-modulatory effects *via GPX4* inhibition. By downregulating *GPX4*, Curcumin provokes autophagy-mediated ferroptosis in non-small cell lung cancer cells, ultimately curbing tumor development [290]. Similarly, *boswellia carterii n-hexane extract (BCHE)* exerts its cytotoxic effects on tumor cells in breast cancer by enhancing transferrin synthesis and Fe²⁺ accumulation, concomitantly repressing *GPX4* to exacerbate ROS-induced lipid peroxidation and ferroptosis [291]. Emerging evidence highlights botanical agents such as baicalein, salidroside, and quercitrin, which disrupt *GPX4*-catalyzed lipid repair pathways to selectively induce cancer cell ferroptosis with negligible toxicity [292–297]. Currently, it has also been found that vitamin K2 shows its anti-osteoarthritis (OA) efficacy through a dual-target regulation mechanism. On the one hand, it proficiently antagonizes the activation of the *MAPK/ NF-κB* signaling cascade – triggered by reduced *GPX4* expression – thereby delaying the catabolic progression of the extracellular matrix. On the other hand, vitamin K₂ mitigates the inhibitory impact of *RSL3* on *GPX4* function, allowing the ferroptosis process to proceed smoothly [298]. Covalent targeting of selenocysteine residues, achieved through alkyl chloride activation, defines the mechanism of existing *GPX4* inhibitors, all of which are alkylating agents. However, the prevailing strategy among existing *GPX4* inhibitors involves alkylation: reactive alkyl chlorides covalently engage selenocysteine residues, which greatly limits the clinical application of most *GPX4* inhibitors. In a pioneering study, Li’s lab reported that treatment with *BSA-SeNPs* powerfully activates *NRF2*, consequently eliciting an increase in the antioxidant enzyme *GPX4* across both its mRNA and protein forms, effectively counteracting ROS accumulation during erastin-triggered ferroptosis [252]. This contributes new perspectives for engineering *GPX4* inhibitors.

### 3.2. Research progress of ferroptosis inhibitors

Ferroptosis is initiated and sustained by two core alterations: massive iron accumulation within the cell, which fuels widespread lipid peroxidation. To prevent this, a class of compounds known as ferroptosis inhibitors is used. They work either by capturing free radicals, blocking the production of lipid peroxides, or removing free iron [299,300]. This diversity in their functional approach leads to their main classification into two categories: iron ion chelators and lipid peroxidation regulators.

#### 3.2.1. Lipid peroxidation regulator

Ferrostatin-1 (Fer-1) is a potent and highly selective blocker of ferroptosis. Data collected from multiple studies validate that Fer-1 blocks iron accumulation in spinal anterior horn neurons by activating the *ERK1/2/Specificity Protein 1 (SP1)/GPX4* signaling cascade. This mechanism plays a significant role in improving neural function injury and blood spinal cord barrier (BSCB) damage in rats after spinal ischemia reperfusion injury (SCIRI). Per experimental studies (*in vivo/in vitro*) by He et al. Fer-1 inhibits angiotensin II-induced ferroptosis in vascular smooth muscle cells (VSMCs) *via SLC7A11/GPX4* pathway activation, thereby delaying abdominal aortic aneurysm (AAA) formation and preserving vascular wall integrity [301]. Within a mouse model system simulating LPS-driven lung injury, Fer-1 exhibits inhibitory effects on lipid peroxidation [301]. Emerging research further reveals that Fer-1 ameliorates neuronal ferroptosis caused by hypoxic-ischemic brain damage (HIBD) by suppressing erastin-driven ROS generation and oxidative stress [302]. Additionally, selective ferroptosis inhibition has been shown to reverse the cytotoxic effects of isoflurane and other anesthetics on astrocytes, alleviating prolonged isoflurane-induced cognitive impairment and potentially addressing neonatal cognitive dysfunction [303]. These findings underscore Fer-1’s robust suppression of lipid peroxidation across diverse ferroptotic stimuli. Structural analogs of Fer-1, such as *SRS11-92* and *SRS11-86*, offer enhanced stability and superior tissue-protective capabilities [304,305]. For instance, *SRS11-92* confers neuroprotection by inhibiting *NRF2*-mediated oxidative stress and ferroptosis [306]. Liproxstatin-1 (Lip-1), a spiroquinoxaline amine compound, reduces ischemia/reperfusion (I/R)-induced acute kidney injury (AKI) primarily by targeting *EGR1* to suppress ferroptosis and subsequent inflammatory factor release [307]. Similarly, Lip-1 can also alleviate ischemia-reperfusion injury induced by lung transplantation by inhibiting ferroptosis [308]. The exacerbation of acute pancreatitis (AP) by hypertriglyceridemia (HTG) is well-documented, particularly through an inflammatory response intrinsically connected to ferroptosis. Targeting this axis, Lip-1 exerts its therapeutic effect *via* lipid metabolism regulation, which restrains ferroptosis and ultimately preserves pancreatic integrity [309]. Furthermore, experimental findings demonstrate that Lip-1 downregulates ferroptosis-related proteins such as *ACSL4* and *TP53*, alongside modulating time-restricted feeding. Concurrently, treatment maintains mitochondrial structural integrity and functional capacity, accompanied by reduced cellular lipid peroxidation and ROS generation [310]. Supporting its cardioprotective role, Lip-1 has been empirically validated to relieve hypertensive heart damage, fibrotic progression, and adverse cardiac remodeling. This protection operates *via* augmented *GPX4* signal transduction, effective ferroptosis clearance, and constrained lipid peroxidation [311,312]. Collectively, these discoveries provide novel insight into ferroptosis regulatory mechanisms and highlight promising targets for developing therapies against ferroptosis-associated pathologies.

#### 3.2.2. Iron ion chelator

Iron chelators exert their primary function by sequestering excess cellular iron. This binding action prevents iron from donating electrons to oxygen species, thereby cutting down on highly reactive hydroxyl radical generation. By curbing this fenton reaction, iron chelators effectively suppress ferroptosis [313–315]. New research has revealed that iron chelators, by targeting viral iron-dependent mechanisms, modulating host immunity, and influencing cell death pathways, have demonstrated broad-spectrum antiviral potential. With advancements in targeted delivery technologies and deeper mechanistic insights, they are poised to become a novel tool in antivirus therapy, particularly in combating drug-resistant viruses or severe infections, where they could play a unique role [316]. Iron chelators have demonstrated significant potential in slowing the neurodegenerative progression of Alzheimer’s Disease (AD) by chelating abnormally deposited iron ions in the brain, inhibiting oxidative stress responses, and reducing pathological protein accumulation, thereby offering a novel therapeutic strategy for AD intervention [317–321]. Cutting-edge research has shown that polydopamine nanoparticles (PDA NPs) offer a promising therapeutic strategy against intervertebral disc degeneration. They combat ferroptosis through a multifaceted approach: they chelate iron ions, scavenge ROS, and inhibit the ubiquitination of *GPX4*. This action leads to the upregulation of cellular antioxidant pathways [322]. Deferoxamine (DFO), an iron chelator with poor membrane permeability, accumulates in lysosomes *via* endocytosis and interacts with them, intercepting iron ions that should be transported to other parts, thereby preventing the generation of lipid active oxygen species [323–325]. Nevertheless, the abbreviated plasma half-life of deferoxamine necessitates repeated dosing regimens, thereby predisposing patients to side effects. To address this issue, a team from Harvard Medical School developed a novel DFO nano chelator that provides sustained release over two weeks [326,327]. Deferiprone (DFP) combats oxidative stress by chelating intracellular iron, promoting disease regression in various pathologies [328–332]. Notably, Ye et al. employed a mouse model to demonstrate DFP’s capacity to inhibit retinal ferroptosis, preserve retinal structure/function, and provide therapy for retinal detachment [333]. Additionally, studies have found that DFP reduces neuronal ferroptosis and alleviates neurological dysfunction through the *N-myc downstream regulates gene-1 (NDRG1)/YAP* signaling pathway, offering a potential non-invasive treatment for patients with brain injury following intracranial hemorrhage [334].

## 4. Perspective

**Figure 2.**
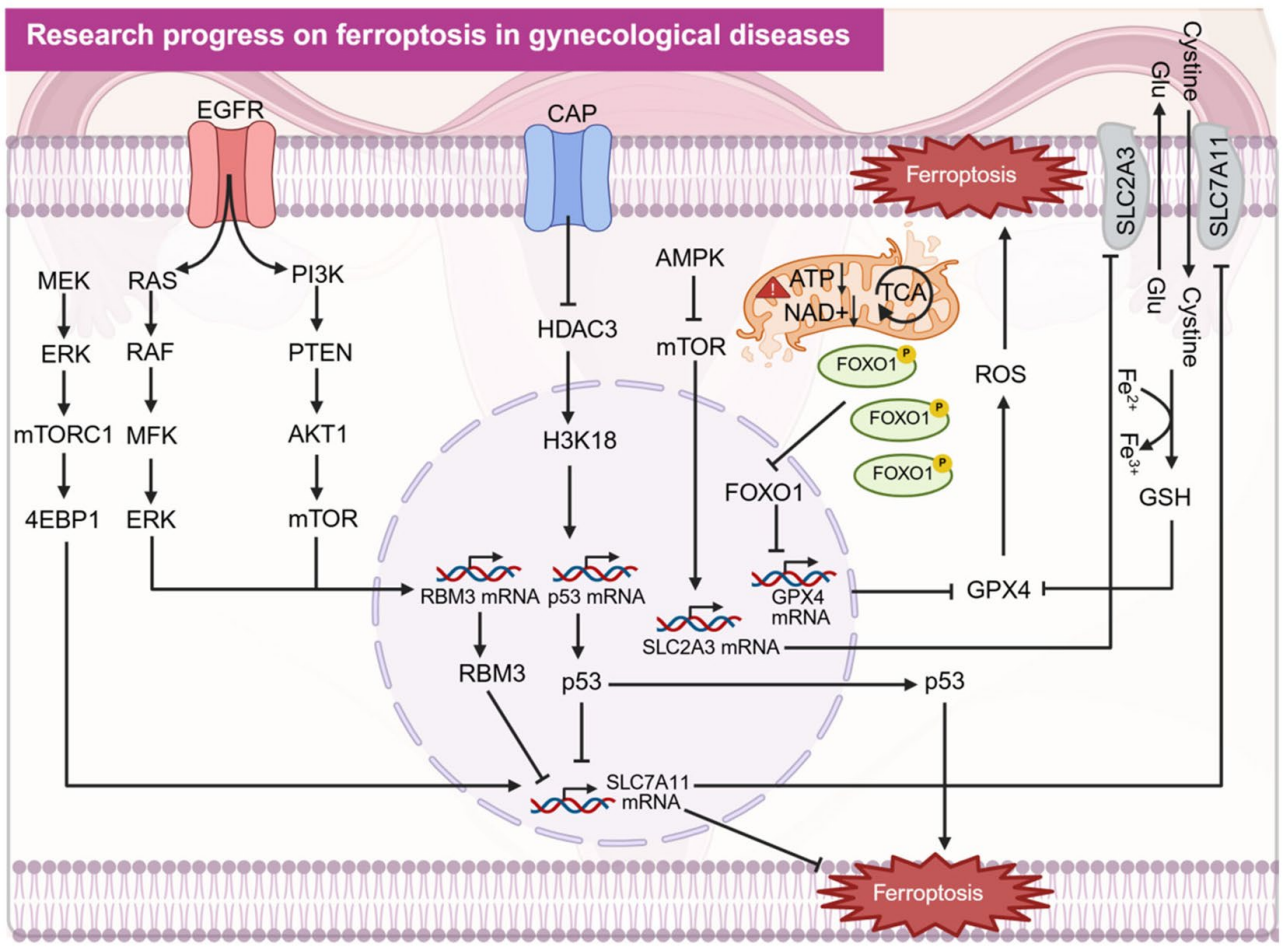
The primary signaling pathways of ferroptosis: System Xc-, a heterodimer consisting of *SLC7A11* and *SLC3A2*, performs a cystine/glutamate antiport function across the plasma membrane. Cystine is vital for GSH synthesis. Inhibiting *GPX4* or having low GSH leads to lipid peroxide buildup, membrane damage, and ferroptosis, releasing ROS and increasing oxidative stress. Genes like *TP53* and *RBM3* inhibit *SLC7A11* expression, promoting ferroptosis.

Recent years have seen a growing recognition of ferroptosis as a clinically significant player in the complex processes underlying numerous diseases. Its role in the development, worsening, and treatment of conditions ranging from neurodegenerative diseases and cancers to strokes, brain trauma, organ injury, and liver and kidney failure is now becoming increasingly clear [335–341]. As research progresses, the important role of ferroptosis in gynecological diseases has increasingly attracted the attention of scientists (Figure 2). However, compared to other diseases, the crucial mechanisms linking ferroptosis to gynecological disorders are not yet well elucidated. This might be limited by the scarcity of *in situ* animal models for gynecological diseases and the relatively weak foundational research in this area. Ferroptosis research in gynecology is currently at an early but promising stage. Unraveling the complex mechanisms and identifying the core regulatory nodes of ferroptosis during the development and advancement of gynecological diseases presents a significant opportunity for both theoretical advancement and clinical innovation. State-of-the-art methodologies, including single-cell RNA sequencing, open chromatin sequencing at the single-cell level, and spatial transcriptomic analysis, are now available to propel this field forward. These technologies help us better understand the ferroptosis related changes at the single cell level in gynecological diseases, thereby accelerating the resolution of key mechanisms of ferroptosis in these conditions [342–348].

Although many basic studies have reported that ferroptosis regulators show certain effects at the cellular and animal levels, these compounds mainly face several problems, such as poor physical and chemical properties, suboptimal pharmacokinetics, characterized by systemic exposure and associated toxicity, rapid clearance from the body, insufficient distribution to target areas, and an inability to exert strong ferroptosis induction or modulation (Table 6). These compounds often have poor draggability, leading to subpar clinical applications. Presently, a significant emphasis in clinical translation lies on the development of agents capable of specifically targeting ferroptosis. In recent years, advances in nanotechnology and related targeted drug delivery modalities have emerged as potent enablers for creating novel ferroptosis regulators. Notably, nano-drug delivery systems effectively overcome pharmacokinetic hurdles, including limited aqueous solubility and impaired membrane penetration, to facilitate site-specific accumulation [349–354]. The strides made hold substantial promise for the eventual clinical deployment of ferroptosis-modulating compounds to combat gynecological disorders.

**Table 6.** Research progress on ferroptosis in gynecological diseases.

Diseases	Prognostic related genes		Pathways	References
	Good	Poor		
EC	*SOX4* *GPX4* *SLC11A2* *SLC7A11* *NRF2* *NF-KB* *SAT1* *TP53* *FOXA2* *CAPG*	*GLP1R* *ACAT2* *LDHA* *CDKN2A* *FZD7* *ACTN4* *MYH10* *LCN2* *hsa-mir-4758* *ENPP2*	*NaBu/RBM3/* *SLC7A11*	[31]
EMS	*BECN1* *GSK3B* *IREB2* *TIGIT* *GPX4* *ACSL4* *FADD* *FLOT1* *HLA-DMA*	*CD24* *FAS* *PIK3CA* *PTPN11* *TGFBR2* *CFL1* *CHMP6* *CISD3*	*lncRNA ADAMTS9-AS1/* *miR-6516-5p/GPX*	[72]
			*lncRNA MALAT1/* *miR-145-5p/MUC1*	[73]
			*miR-21-3p/* *p53/SLC7A11*	[74]
			*p38/JNK/ATF3/* *SLC7A11*	[76,77]
PCOS	*GSH* *GPX4* *TFR1* *FTH1* *ATF3* *DDIT4* *LPIN1* *NOS2* *NQO1* *SLC2A1* *SLC2A6*	*JUN* *STAT3* *HMOX1* *HMGB1* *PPAR-α* *BCAP31* *EDEM1* *TRIB3* *ERMP1*	*PER1/SREBF2/* *ALOX15*	[96]
			*SIRT3/AMPK/* *mTOR*	[97]
			*METTL3/m6A/* *GPX4*	[99]
			*ATR/PDK4/* *JAK/STAT3*	[100]
			*AMPK/NRF2*	[101]
			*p38/JNK/SLC7A11*	[102]
			*YAP1/NRF2*	[104]
			*circ_0097636/* *miR-186-5p/SIRT3*	[108]
			*SCM − 198/* *SLC7A11/GPX4*	[109]
			*CD44/SLC7A11*	[110]
OC	*ALOX12* *STUB1* *LAG3* *TIGIT* *CTLA4* *IDO1* *CD27* *ICOS* *IL2RB*	*SLC7A11* *SOX2* *FH* *GCH1* *MYCN* *FURIN* *SQLE* *PARK7* *HOXB3* *PVR*	*JAM3/NRF2/FSP1*	[133]
			*OSGIN1/ATM/* *AMPK/SLC2A3*	[136]
			*SB/ARHGAP10/* *GPX4*	[137]
			*STEAP3/p53/* *SLC7A11*	[138]
			*lncRNA TPT1-AS1/* *GPX4/KHDRBS3*	[139]
			*SGK1/mTOR/* *SREBP1/SCD1*	[141]
			*mTOR/eIF4E/* *SLC7A11*	[143]
			*SPHK1/* *NF-κB/NRF2*	[148]
			*Ca2+/CAMKK2/* *AKT/mTOR*	[149]
			*NAT10/ACOT7*	[150]
CC	*SLC7A5* *TAZ* *SOX2* *KLF14* *MPC1* *FBXW7* *G6PD* *TP53* *ZNF419* *TFRC*	*ANO6* *KDM3A* *ANGPTL4* *P4HA1* *PGK1* *VEGFA* *TFRC* *CNIH4* *GPX4* *SLC7A11* *SLC2A14* *SQLE*	*miR-30c-5p/* *METTL3/KRAS*	[180]
			*METTL3/COTE-1*	[181]
			*METTL14/FTH1/* *PI3K/AKT*	[182]
			*EPAS1/MALAT1-Mir-182-5p-EPAS1*	[183]
			*MTCH1/FOXO1/* *GPX4*	[184]
			*p62/ KEAP1 /NRF2* *NRF2/GPX4/xCT*	[185]
			*CENPF/NRF2/HO-1*	[186]
			*USP34/cGAS/* *STING*	[191]
			*CBP/p300/TUBORF*	[192]
			*NRF2/GPX4/xCT*	[194]
			*PI3K/AKT/MTOR*	[195]

## Data Availability

Data sharing is not applicable to this article as no new data were created or analyzed in this study.

## Abbreviations

ACAT2: Acetyl-CoA Acetyltransferase 2
ACSL4: Acyl-CoA Synthetase Long Chain Family Member 4
ACTN4: Actinin Alpha 4
ALOX12: Arachidonate 12-Lipoxygenase
ANGPTL4: Angiopoietin Like 4
ANO6: Anoctamin 6
ATF3: Activating Transcription Factor 3
BCAP31: B Cell Receptor Associated Protein 31
BECN1: Beclin 1
BRCA1: Breast cancer type 1
CAPG: Capping Actin Protein, Gelsolin Like
CAV1: Caveolin 1
CDKN1A: Cyclin Dependent Kinase Inhibitor 1 A
CDKN2A: Cyclin Dependent Kinase Inhibitor 2 A
CFL1: Cofilin 1
CHMP6: Charged Multivesicular Body Protein 6
CISD1: CDGSH iron-sulfur domain-containing protein 1
CISD3: CDGSH Iron Sulfur Domain 3
CNIH4: Cornichon Family Member 4
CTLA4: Cytotoxic T-Lymphocyte Associated Protein 4
DDIT4: DNA Damage Inducible Transcript 4
EDEM1: ER Degradation Enhancing Alpha-Mannosidase Like Protein 1
ENPP2: Ectonucleotide Pyrophosphatase 2
EPAS1: Endothelial PAS Domain Protein 1
ERMP1: Endoplasmic Reticulum Metallopeptidase 1
FADD: Fas Associated via Death Domain
FANCD2: FA Complementation Group D2
FAS: Fas Cell Surface Death Receptor
FBXW7: F-Box And WD Repeat Domain Containing 7
FH: Fumarate Hydratase
FLOT1: Flotillin 1
FOXA2: Forkhead Box A2
FTH1: Ferritin Heavy Chain 1
FURIN: Furin, Paired Basic Amino Acid Cleaving Enzyme
FZD7: Frizzled Class Receptor 7
G6PD: Glucose-6-Phosphate Dehydrogenase
GCH1: GTP Cyclohydrolase 1
GCLC: Glutamate-Cysteine Ligase Catalytic Subunit
GLP1R: Glucagon Like Peptide 1 Receptor
GSK3B: Glycogen Synthase Kinase 3 Beta
HLA-DMA: Major Histocompatibility Complex, Class II, DM Alpha
HMGB1: High Mobility Group Box 1
HOXB3: Homeobox B3
HSBP1: Heat shock factor-binding protein 1
ICOS: Inducible T Cell Costimulator
IDO1: Indoleamine 2,3-Dioxygenase 1
IL2RB: Interleukin 2 Receptor Subunit Beta
IREB2: Iron Responsive Element Binding Protein 2 ().
JUN: Jun Proto-Oncogene
KDM3A: Lysine Demethylase 3 A
KLF14: KLF Transcription Factor 14
LAG3: Lymphocyte Activating 3
LCN2: Lipocalin 2
LDHA: Lactate Dehydrogenase A
LPIN1: Lipin 1
MAP3K5: Mitogen-Activated Protein Kinase Kinase Kinase 5
MPC1: Mitochondrial Pyruvate Carrier 1
MYCN: N-myc proto-oncogene protein
MYH10: Myosin Heavy Chain 10
NF-κB: Nuclear Factor Kappa B Subunit
NOS2: Nitric Oxide Synthase 2
NQO1: NAD(P)H Quinone Dehydrogenase 1
NRF2: Nuclear factor erythroid 2-related factor 2
P4HA1: Prolyl 4-Hydroxylase Subunit Alpha 1
PARK7: Parkinson disease protein 7
PGK1: Phosphoglycerate Kinase 1
PIK3CA: Phosphatidylinositol-4,5-Bisphosphate 3-Kinase Catalytic Subunit Alpha
PPAR-α: Peroxisome Proliferator Activated Receptor Alpha
PTPN11: Protein Tyrosine Phosphatase Non-Receptor Type 11
PVR: Poliovirus receptor
SAT1: Serine acetyltransferase 1
SAT1: Spermine N1-Acetyltransferase 1
SLC11A2: Solute Carrier Family 11 Member 2
SLC2A1: Solute Carrier Family 2 Member 1
SLC2A14: Solute Carrier Family 2 Member 14
SLC2A6: Solute Carrier Family 2 Member 6
SLC7A11: Solute Carrier Family 7 Member 11
SLC7A5: Solute Carrier Family 7 Member 5
SOX2: SRY-Box Transcription Factor 2
SOX4: SRY-Box Transcription Factor 4
SQLE: Squalene Epoxidase
STUB1: STIP1 Homology And U-Box Containing Protein 1
TAZ: Tafazzin
TFR1: Transferrin Receptor 2
TFRC: Transferrin Receptor
TGFBR2: Transforming Growth Factor Beta Receptor 2
TIGIT: T Cell Immunoreceptor With Ig And ITIM Domains
TIGIT: T Cell Immunoreceptor With Ig And ITIM Domains
TRIB3: Tribbles Pseudokinase 3
VEGFA: Vascular Endothelial Growth Factor A
ZNF419: Zinc Finger Protein 419
